# Magnitude and predictors of normal-weight central obesity– the AWI-Gen study findings

**DOI:** 10.1080/16549716.2019.1685809

**Published:** 2019-11-07

**Authors:** Shukri F. Mohamed, Tilahun Nigatu Haregu, Christopher Khayeka-Wandabwa, Stella Kagwiria Muthuri, Catherine Kyobutungi

**Affiliations:** a Health and Systems for Health Unit (HSH), African Population and Health Research Center (APHRC), Nairobi, Kenya; b Division of Health Sciences, Warwick Medical School, University of Warwick, Coventry, UK; c Non-Communicable Disease Unit, School of Population and Global Health, University of Melbourne, Melbourne, Australia; d School of Pharmaceutical Science and Technology (SPST), Health Science Platform, Tianjin University, Tianjin, China

**Keywords:** Central obesity, normal-weight, informal settlement, slums, Africa

## Abstract

**Background**: Normal-weight central obesity is associated with higher mortality than general obesity as defined by body mass index, particularly in the absence of central fat distribution.

**Objective**: The aim of this study was to examine the magnitude and predictors of normal-weight central obesity in an urban informal settlement setting in Kenya.

**Methods**: We used data from the AWI-Gen study, a cross-sectional survey targeting randomly selected consenting adults between the ages of 40–60 in two urban informal settlements of Nairobi between 2014 and 2016. Central obesity was determined using waist circumference, waist to hip ratio, visceral fat thickness, and subcutaneous fat thickness. General obesity was determined using body mass index (BMI).

**Results**: About 20.0% of participants in the study had general obesity. The prevalence of central obesity as measured by waist circumference was 52.0%, by waist-to-hip ratio was 53.5%, by visceral fat thickness was 32.4% and by subcutaneous fat thickness was 49.2%. The prevalence of normal-weight central obesity in the study population was highest when measured by waist to hip ratio (38.1%) and lowest when measured by visceral fat thickness (18.1%). Factors associated with normal-weight central obesity as assesses by waist circumference were being female, of older age, and in full-time employment. Older age was associated with normal-weight central obesity as assessed by waist to hip ratio.

**Conclusion**: The findings highlight a significant prevalence of normal-weight central obesity among adults in a poor urban setting in Kenya, pointing to women as a key target group for focused interventions. Longitudinal studies are needed to establish whether there is a link between normal-weight central obesity and mortality in such settings as has been found in other settings.

## Background

Obesity is considered a key risk factor for many non-communicable diseases (NCDs) including cardio-metabolic diseases, chronic respiratory disease, osteoarthritis, and certain cancers. Accordingly, globesity, a global epidemic of obesity and overweight has been a critical challenge for global and national level health systems. Previously, obesity was considered a problem for developed countries; however, current research shows that obesity is increasing in low and middle-income countries, including in poor urban settings [1–3].

By 2014, about 13% of the world’s adult population (11% of men and 15% of women) and 39% of adults aged 18 years and over (38% of men and 40% of women) were obese and overweight, respectively. Nearly two billion adults aged 18 years and older were overweight, and of those, over 600 million adults were obese. The worldwide prevalence of obesity has grown by more than two-fold during the last 35 years. Many low- and middle-income countries, which are still struggling to address the problems of under-nutrition, are already facing a rapid upsurge of obesity and overweight, particularly in their urban settings. In many urban settings of these countries, it is now common to find co-existing obesity and under-nutrition in the same community and even within the same household [4–6].

Consistently strong relationships have been observed between obesity and risk of developing cardio-metabolic diseases [7–9]. Normal-weight central obesity is defined as having normal weight using Body Mass Index (BMI) calculated from weight (kg)/height (m^2^) and central obesity using various anthropometric indicators such as waist circumference (WC), waist-hip ratio (WHR), visceral fat thickness (VCT) and subcutaneous fat thickness (SCFT) among other measures [10]. Recent studies have shown that normal-weight central obesity is associated with higher mortality than general obesity as defined by BMI only [11,12]. Consequently, individuals with normal-weight central obesity have lower long-term survival. Previous studies have also shown that men and women with a normal-weight central obesity as measured by WHR, had 1.87 and 1.48 times greater total mortality risk than those with similar BMI but no central obesity [12]. Further, men and women with normal-weight central obesity have 2.24 and 1.32 times the mortality risk of participants who were overweight or obese according to BMI only [12]. As a result, central obesity in normal-weight people places them at greater risk of death than does overall overweight or obesity in people without the excess abdominal fat [12–14]. However, evidence about the magnitude and determinants of normal-weight central obesity is limited in informal settlement settings in sub-Saharan Africa (SSA).

The aim of this paper was to examine the prevalence and predictors of normal-weight central obesity among urban informal settlement dwellers in Nairobi, Kenya. More specifically, the paper aimed to determine and compare the magnitude of normal-weight central obesity by using different measures of central obesity. Also, of interest is how the type of predictors and their effects vary when different measurements of central obesity are used.

## Methods

### Data source, study population and sampling

Data for this study were obtained from the AWI-Gen study (Africa Wits-INDEPTH Partnership for the Genomic Research) – Human Heredity and Health in Africa (H3Africa) study. This was a cross-sectional household survey involving five health and demographic surveillance system field sites within the INDEPTH Network across four countries, Ghana, Burkina Faso, Kenya, and South Africa. The aim of the overall study was to provide estimates for indicators on NCDs risk factors for persons aged 18–69 years, and to identify genetic factors that contribute to body composition, including obesity, which together with environmental factors, increase susceptibility to cardio-metabolic disease [15–17]. The AWI-Gen study protocol including participant recruitment/enrolment, inclusion/exclusion criteria, sample size determinations is available elsewhere in detail [16,17]. The analyses presented herein focus solely on the data collected from 2003 adults aged 40–60 years [18,19] in the Nairobi Urban Health and Demographic Surveillance System, a pioneer urban-based Health and Demographic Surveillance system in SSA as earlier described in detail [20].

### Measurements

Data on weight, height, waist circumference, hip circumference, and abdominal subcutaneous fat were collected from 2003 adults in Korogocho and Viwandani; two urban slums of Nairobi, between 2014 and 2016. WC was measured using SECA 201 circumference measurement tape. A LOGIQ e ultrasound system with a 2–5 MHz 3C-RS curved array transducer was used to determine abdominal adipose depths as proxies for visceral and subcutaneous adipose tissue.

### Cut-off points for obesity

Cut-off points were defined based on World Health Organization recommendations [10,21]. General obesity was defined by body mass index (BMI) of ≥30 kg/m^2^ and overweight was defined by BMI greater than or equal to 25 kg/m^2^. Central obesity was determined using four measures: WC >94 cm for men and >80 cm for women, WHR >0.90 for men and >0.85 for women [10], and VFT > 6.5 cm for men and >5 cm for women and SCFT >1.5 cm [22,23]. Normal-weight central obesity using WC as a measure is defined as having a normal BMI and WC >94 cm for men and >80 cm for women. Normal-weight central obesity using WHR as a measure is defined as having a normal BMI and a WHR >0.90 for men and >0.85 for women.

### Data analysis

Data were analyzed using Stata 13.0. The prevalence of general obesity, central obesity, and normal-weight central obesity were described using proportions. Predictors of normal-weight central obesity were identified using multiple logistic regression analysis by using WC and WHR as measurers of central obesity separately. P-values greater than 0.05 were considered to be statistically significant.

## Results

### Characteristics of study population

A total of 2003 adults participated in the study. Of these, the majority (54%) were women, 62.6% were aged 40–50 years, 57.5% had primary level of education as their highest level of education, 36.2% were from the Kikuyu ethnic group and 47.3% were self-employed. The background characteristics of the participants are presented in Table 1.

**Table 1.** Background characteristics of the study population.

	Categories	Women n (%)	Men n (%)	Total n (%)	P-value
Age groups	40–45 years	397 (54.8%)	327 (45.2%)	724 (36.1%)	0.012
	46–50 years	308 (58.1%)	222 (41.9%)	530 (26.5%)	
	51–55 years	230 (52.8%)	206 (47.2%)	436 (21.8%)	
	56–60 years	146 (46.7%)	167 (53.3%)	313 (15.6%)	
Education	No formal education	118 (76.6%)	36 (23.4%)	154 (7.7%)	0.000
	Primary education	682 (59.3%)	469 (40.8%)	1151 (57.5%)	
	Secondary education	277 (41.2%)	395 (58.8%)	672 (33.5%)	
	Tertiary education	4 (15.4%)	22 (84.6%)	26 (1.3%)	
Ethnicity	Kamba	196 (49.9%)	197 (50.1%)	393 (19.6%)	0.000
	Kikuyu	480 (66.2%)	245 (33.8%)	725 (36.2%)	
	Luhya	143 (44.4%)	179 (55.6%)	322 (16.1%)	
	Luo	159 (42.5%)	215 (57.5%)	374 (18.7%)	
	Others	103 (54.5%)	86 (45.5%)	189 (9.4%)	
Employment	Self-employed	632 (66.8%)	314 (33.2%)	946 (47.3%)	0.000
	Employed (Full-time)	50 (18.7%)	217 (81.3%)	267 (13.4%)	
	Employed (Part-time)	19 (42.2%)	26 (57.8%)	45 (2.3%)	
	Employed (Informal)	287 (46.1%)	336 (53.9%)	623 (31.2%)	
	Unemployed	93 (78.2%)	26 (21.8%)	119 (5.9%)	
	**Total**	**1,081 (54.0%)**	**922 (46.0%)**	**2003 (100.0%)**	

### General obesity

The overall prevalence of general obesity as defined by BMI of >30 kg/m^2^ was generally distributed among the different age groups as shown in Table 2. Overall, women had significantly higher general obesity compared to men (32.2% vs. 5.6%; p < 0.001). Similar patterns were seen across all the age-groups, where women had a significantly higher prevalence of general obesity compared to men in the respective age-groups. About one in four people were overweight (BMI > 25 kg/m^2^ but less than 30 kg/m^2^). Of the study population, 942 (47.0%) had normal BMI and 150 (7.5%) were underweight. Among women, prevalence of general obesity increased with age and was highest among women aged 56–60 (36.3%).

**Table 2.** Prevalence of general obesity in the study population by age and sex.

Age groups	Women	Men	Total	P-value
40–45 years	123 (31.0%)	20 (6.1%)	143 (19.8%)	<0.001
46–50 years	93 (30.2%)	4 (1.8%)	97 (18.3%)	<0.001
51–55 years	79 (34.4%)	12 (5.8%)	91 (20.9%)	<0.001
56–60 years	53 (36.3%)	16 (9.6%)	69 (22.0%)	<0.001
Total	348 (32.2%)	52 (5.6%)	400 (20.0%)	<0.001

### Central obesity

Based on the International Diabetes Federation recommended cut-off points for waist circumference [10,21] (>94 cm for men and >80 cm for women), as shown in Table 3, the overall prevalence of central obesity was 52.0% (75.6% among women and 24.4% among men). The prevalence of central obesity increased with increasing age and this was statistically significant (P < 0.001). Like the general obesity, central obesity was also higher among those 51–60 years than in those 40–50 years (50.0% and 56.0%, respectively).

**Table 3.** Prevalence of central obesity by age category and gender.

	Age groups	Women n (%)	Men n (%)	Overall n (%)	P-Value
Waist Circumference	40–45 years	275 (69.3%)	70 (21.4%)	345 (47.6%)	<0.001
	46–50 years	232 (75.3%)	46 (20.7%)	278 (52.4%)	<0.001
	51–55 years	182 (83.5%)	47 (22.8%)	239 (54.8%)	<0.001
	56–60 years	118 (80.8%)	62 (37.1%)	180 (57.5%)	<0.001
	Total	817 (75.6%)	225 (24.4%)	1,042 (52.0%)	
Waist-to-hip ratio (WHR)	40–45 years	218 (54.9%)	108 (33.0%)	326 (45.0%)	<0.001
	46–50 years	199 (64.6%)	85 (38.3%)	284 (53.6%)	<0.001
	51–55 years	170 (73.9%)	87 (42.2%)	257 (58.9%)	<0.001
	56–60 years	117 (80.1%)	88 (52.7%)	205 (65.5%)	<0.001
	Total	704 (65.1%)	368 (39.9%)	1072 (53.5%)	
Visceral Fat Thickness (VFT)	40–45 years	151 (38.0%)	64 (19.6%)	215 (29.7%)	<0.001
	46–50 years	119 (38.6%)	41 (18.6%)	160 (30.1%)	<0.001
	51–55 years	109 (47.4%)	52 (25.2%)	161 (36.9%)	<0.001
	56–60 years	65 (44.5%)	47 (28.1%)	112 (35.8%)	0.001
	Total	444 (41.1%)	204 (22.1%)	648 (32.4%)	
Subcutaneous Fat Thickness (SCT)	40–45 years	271 (68.3%)	78 (24.0%)	349 (48.3%)	<0.001
	46–50 years	217 (70.5%)	43 (19.4%)	260 (49.1%)	<0.001
	51–55 years	173 (75.5%)	47 (22.8%)	220 (50.5%)	<0.001
	56–60 years	104 (71.2%)	52 (31.1%)	156 (49.8%)	<0.001
	Overall	765 (70.8%)	220 (23.9%)	985 (49.2%)	
	Total (N)	1081	922	2003	

n = number of people with central obesity, % = percent with central obesity among same gender and age group, N = total sample size, p-values = are from chi-square test of association between central obesity and gender within each age category.

Using WHR cut-off points (>0.9 for men and >0.85 for women), the prevalence of central obesity in the study population was 53.5% (39.9% in men and 65.1% in women). The prevalence among those 40–50 years and 51–60 years of age were 48.6% and 61.7%, respectively.

The overall prevalence of central obesity as measured by VFT was 32.4% (22.1% among men and 41.1% among women). It was 29.9% among those 40–50 years and 36.5% among 51–60-year-old participants. The prevalence of central obesity using SCFT of >1.5 cm was 49.2% (23.9% among men and 70.8% among women). The age variation for this was minimal.

All measures of central obesity consistently showed a high prevalence of overweight and obesity in the study population. In all the measurements, women and older adults had higher burden of central obesity. In all of these measures, at least one in three adults (one in five men and two in five women) were found to be centrally obese.

### Normal-weight central obesity

On the basis of the comparison between BMI and WC, the overall prevalence of normal-weight central obesity was 21.2%. This was 44.7% in women and 6.7% in men as shown in Table 4. The prevalence among those 40–50 and 51–60 years old was 18.5% and 26.0%, respectively. The prevalence of normal-weight central obesity, using WHR as a measure of central obesity, was 38.1% (30.4% in men and 50.6% in women). When measurements of abdominal fat thickness were used to define central obesity, the prevalence of normal-weight central obesity were 18.1% for VFT and 23.6% for SCFT. Overall, the prevalence of normal-weight central obesity ranges from 18.1% to 38.1% depending on the types of measure used for central obesity. In all of these measures, at least one in five adults had normal weight central obesity.

**Table 4.** Prevalence of central obesity by BMI categories and gender.

	BMI status	Women n (%)	Men n (%)	Total n (%)	P-Value
Waist circumference (WC)	Underweight	3 (7.3%)	1 (0.9%)	4 (2.67%)	<0.001
	Normal BMI	161 (44.7%)	39 (6.7%)	200 (21.2%)	<0.001
	Overweight	306 (92.2%)	134 (74.9%)	440 (86.1%)	<0.001
	Obesity	347 (99.7%)	51 (98.1%)	398 (99.5%)	0.115
	Total	817 (75.6%)	225 (24.4%)	1042 (52.0%)	
Waist-to-hip-ratio (WHR)	Underweight	15 (36.6%)	14 (12.8%)	29 (19.3%)	<0.001
	Normal BMI	182 (50.6%)	177 (30.4%)	359 (38.1%)	<0.001
	Overweight	233 (70.2%)	130 (72.6%)	363 (71.0%)	0.539
	Obesity	274 (78.7%)	47 (90.4%)	321 (80.3%)	0.010
	Total	704 (65.1%)	368 (39.9%)	1072 (53.5%)	
Visceral Fat thickness (VFT)	Underweight	3 (7.3%)	4 (3.7%)	7 (4.7%)	0.208
	Normal BMI	74 (20.6%)	96 (16.5%)	170 (18.1%)	0.121
	Overweight	139 (41.9%)	62 (34.6%)	201 (39.3%)	0.081
	Obesity	228 (65.5%)	42 (80.8%)	270 (67.5%)	0.021
	Total	444 (41.1%)	204 (22.1%)	648 (32.4%)	
Subcutaneous Fat thickness (SCFT)	Underweight	2 (4.8%)	1 (0.9%)	3 (2.0%)	0.021
	Normal BMI	143 (39.7%)	79 (13.6%)	222 (23.6%)	<0.001
	Overweight	281 (84.6%)	107 (59.8%)	388 (75.9%)	<0.001
	Obesity	339 (97.4%)	33 (66.0%)	372 (93.5%)	<0.001
	Total	765 (70.8%)	220 (23.9%)	985 (49.2%)	

n = number of people with central obesity by BMI categories and gender, % is the percent with central obesity by gender and BMI status, p-values = are from chi-square test of association between central obesity and gender within each BMI category.

### Predictors of normal weight central obesity

Given the high correlation between the measures of central obesity, we only report on normal-weight central obesity as measured by WC and WHR in the models used for the identification of predictors. Sex, age, educational status, ethnicity, employment, current use of alcohol, fruit/vegetable intake diet for at least 5 days a week, current smoking, and work condition involving mostly sitting were included in the analysis based on prior knowledge of associated factors. As shown in Table 5, women had an almost five times higher odds of having normal-weight central obesity as measured by WC compared to men. Participants aged 51–60 years had 1.5 times higher odds of having normal-weight central compared to those 40–50 years. Having full-time employment and belonging to the ‘other’ ethnicity category were also associated with a high prevalence of normal-weight central obesity as measured by WC. None of the four behavioral risk factors were associated with normal-weight central obesity as measured by WC and WHR.

**Table 5.** Predictors of normal-weight central obesity as measured by WC and WHR.

	WC			WHR		
	OR	95% CI		OR	95% CI	
**Socio-demographic factors**						
Sex (ref = men)	**4.93**	3.28	7.41	0.92	0.71	1.18
Age (ref = 40–50 years)	**1.48**	1.08	2.03	**1.53**	1.21	1.95
**Education (ref = no education)**						
Primary	0.84	0.51	1.39	1.05	0.66	1.65
Secondary	0.84	0.48	1.46	1.03	0.64	1.67
Tertiary	0.57	0.07	4.7	2.42	0.91	6.43
**Ethnicity (ref = Kamba)**						
Kikuyu	1.19	0.76	1.86	0.68	0.49	0.94
Luhya	0.73	0.39	1.34	0.89	0.61	1.29
Luo	1.47	0.88	2.44	0.75	0.52	1.08
Others	**2.57**	1.49	4.43	0.98	0.63	1.52
**Employment (Ref = self-employed)**						
Employed (Full-time)	**2.1**	1.23	3.57	0.92	0.62	1.36
Employed (Part-time)	1.63	0.6	4.42	1.03	0.46	2.27
Employed (Informal)	1.11	0.77	1.6	1.29	0.98	1.7
Unemployed	0.77	0.4	1.46	1.21	0.74	1.97
**Behavioral factors**						
Current use of alcohol	0.64	0.22	1.82	1.14	0.69	1.88
Currently smoking	1.08	0.39	3.01	1.43	0.86	2.35
At least 5 days fruit or veg	0.77	0.28	2.16	1.15	0.71	1.86
Work involves siting most of time	2.57	0.98	6.74	1.41	0.91	2.19

OR-Odds ratio, WC-Waist circumference, WHR-Waist to hip ratio, 95% CI – 95% confidence interval.

## Discussion

This study provides important estimates for the burden of and factors associated with normal-weight central obesity in two urban informal settlement communities in Nairobi under the AWI-Gen study [18], using different measures of central obesity. The results show a high burden of general obesity and a higher burden of normal-weight central obesity particularly among older women in the communities suggesting the need for targeted interventions focusing on this vulnerable population.

The prevalence of general obesity (BMI >30 kg/m2) and normal-weight central obesity is much higher in women compared to men. In the current sample, general obesity was over 5 times higher in women (32.2%) compared to men (5.6%). These findings are considerably higher than the national figures in Kenya which found obesity was 3 times higher in women than in men [24] but comparatively similar to observations in South Africa on the prevalence and correlates of central obesity and normal-weight central obesity among adults [25]. A growing body of literature demonstrates the increasing burden of general obesity in diverse settings in Kenya and across Africa [1,26–28] but there is scant evidence highlighting the burden of normal-weight central obesity in the region [25]. Normal-weight central obesity research is currently gaining attention in the SSA region.

Considering the study target population is of very low socioeconomic standing, with individuals less likely to afford expensive treatment, the findings from this study setting are concerning. A high proportion of this population group has no health insurance and face catastrophic health expenditure when serious health concerns arise. They are also less likely to adopt and practice recommended lifestyle modification interventions that can prevent obesity and its complications [29–31].

There is increasing evidence showing that normal-weight central obesity is associated with higher risk of all-cause cardiovascular disease and cancer mortality. Central obesity has gained recognition as an independent risk factor for cardio-metabolic diseases and a better predictor of cardiovascular risk than overall obesity [25,32]. Independent of BMI, or even among individuals with normal weight (BMI, <25.0), those with central obesity remain predisposed to increased risk of mortality because of excessive abdominal fat accumulation [12–14,32,33]. Recent obesity management guidelines are aware of this available evidence and are recommending measuring central obesity among people who are overweight (class I obesity) [25,32].

In the current study, the prevalence of normal-weight central obesity was high, ranging from 18.1% to 38.1% using different measures of central obesity. There are several possible explanations for this finding in these settings. Many developing countries are experiencing an epidemiological transition – moving from a high prevalence of infectious disease to a high prevalence of chronic and degenerative diseases [34]. This change is largely related to shifts in dietary patterns among other health-behaviors, including a decline in physical activity, increased alcohol consumption and tobacco use over the last few decades. For instance, diets in developing countries have rapidly changed to diets high in saturated fat, caloric sugars, salt, refined foods and foods low in fiber often referred to as the Western diet [35]. This shift in consumption patterns, coupled with lifestyle changes in developing countries has resulted in a rapid increase in NCDs and their risk factors [36]. Central obesity – a factor of metabolic syndrome has been identified as a threat to the cardio-metabolic health of individuals. Central obesity plays a crucial role in the pathogenesis of cardiovascular diseases and certain cancers by stimulating mediating factors such as insulin resistance, dyslipidaemia, and systematic inflammation, even among individuals with normal weight [32,33]. A recent study showed that men with normal-weight central obesity as measured by WHR had 1.87 times higher total mortality risk compared to peers with similar BMI and no central obesity, and 2.24 times higher total mortality risk than peers who were overweight or obese as measured by BMI alone [12]. Similar observations were made among women in the same study [12]. If central obesity in normal-weight people places individuals at a greater risk of the underlying pathogenesis resulting in morbidity and mortality than overall overweight or obesity in people without the excess central adiposity, then our finding of proportions of between 18.1% and 38.1% are indeed concerning. The magnitude and burden reported in this study calls for further follow investigation using a longitudinal cohort. This will provide an opportunity to explore the link between normal-weight-central-obesity and mortality in African populations. The addition of anthropometric indicators of central obesity to BMI in all clinical assessments has been recommended [25,32] and should be included in resource poor settings.

Similarly, across all measures of central obesity, women and those in the older age-group categories had higher measures of central obesity compared to men and those in the younger age-groups. Women consistently had higher proportions of normal-weight central obesity, compared to men. Our findings confirm what has been reported in other studies that applied various measures of central obesity [37,38]. Likely reasons for this observation include that women are physiologically more predisposed to overweight and obesity due to the changes occurring during the reproductive years [39], and women have been reported to engage in less physically strenuous/demanding activities in the moderate to vigorous range [40] compared to men in the same setting. Older aged participants in our study had higher odds of normal-weight central obesity compared to the younger aged participants. Our findings are similar to what has been reported elsewhere [37,38]. This finding is somewhat expected in this age group and is likely due to older adults being engaged in less physically strenuous/demanding activities in the moderate to vigorous range, without a similar decrease in caloric consumption.

Full-time employment was associated with higher odds of normal-weight central obesity probably as a result of their higher likelihood of being in sedentary jobs with access to unhealthy processed foods close to their place of employment [40]. A recent study looking at patterns of work linked to physical activity and sedentary behavior among male and female participants from the same study setting found that vigorous to moderate work-related physical activities, walking or cycling as well as vigorous to moderate intensity sports, were generally higher among men than women [40]. It is important to note that only a small proportion of the study participants were employed on full-time basis and the study sample may not be representative of the larger full-time employed population. Further studies with comparable study populations in the various employment categories are needed.

Our findings therefore demonstrate that normal weight (using BMI) and central obesity can co-exist in poor informal settings and that women and those in older age-groups are mostly affected. The coexistence observed is in tandem with prior reporting in the same study setting where a strong shared inter-linkage between obesity and related non-communicable diseases clinical risk factors – hypertension, diabetes, and dyslipidemia has been established [40] thus suggestive of a population that is more vulnerable not only to overall obesity but also other health risks linked to central obesity [25]. Significant associations in the current study did not apply equally to WC and WHR. WC is an absolute measure of central obesity and is affected by overall body architecture. WHR is a relative measure that considers waist circumference relative to hip circumference. A proportionally higher hip circumference could provide a falsely lower estimate of central obesity. Hence, measurement of central obesity needs to consider multiple measures including WC and WHR. Taken together the above results have implications on obesity care programming particularly in informal settlement settings. More target-specific campaigns which provide screening and counselling opportunities with a core focus on older people, particularly women, are needed.

This study has both strengths and limitations. This is the first study, to the best of our knowledge, to estimate the prevalence and factors associated with normal-weight central obesity among urban informal dwellers in Nairobi, Kenya, using different measures of central obesity. However, the study was of a cross-sectional design thus ruling out casual associations. Further, the current study population was limited to those aged between 40 and 60 thus not representing the general population in informal settlements. Therefore, the findings of this study should be interpreted with the above limitations in mind.

## Conclusion

This study has important policy implications. There is a large proportion of people with normal-weight central obesity living in informal settlements. Thus, clinical evaluations should consider including anthropometric measures in the assessment of excessive body weight rather than basing it on BMI alone. Our findings demonstrate that normal weight and central obesity can co-exist in a poor slum setting and that women and those in older age-groups are mostly affected. Thus, interventions and programs for obesity management and prevention should also target individuals with normal-weight central obesity.

Further research is needed to establish whether there is a link between normal-weight central obesity and mortality in this population. Interventions aimed at battling this high risk group of normal-weight individuals with high levels of central adiposity must include an educational component aimed at debunking beliefs around the risks associated with central obesity even among people who do not appear to be carrying excess weight, and should target women, older-age persons, and include a component that seeks to reduce the risks related with full-time employment, possibly due to sedentary lifestyles.

## Paper context

Obesity is a key risk factor for many non-communicable diseases and it is becoming a growing threat in low and middle-income countries. Recent evidence shows that normal-weight central obesity is associated with higher mortality than general obesity as defined by body mass index (BMI) only. However, this evidence is lacking in resource limited slum settings in sub-Saharan Africa. Therefore, the results of this study are important in guiding interventions aimed at battling normal-weight central obesity.
